# Similarities in the immunoglobulin response and V_H _gene usage in rhesus monkeys and humans exposed to porcine hepatocytes

**DOI:** 10.1186/1471-2172-7-3

**Published:** 2006-03-20

**Authors:** Joanne L Zahorsky-Reeves, Clare R Gregory, Donald V Cramer, Insiyyah Y Patanwala, Andrew E Kyles, Dominic C Borie, Mary K Kearns-Jonker

**Affiliations:** 1 Cardiothoracic Surgery Research, The Saban Research Institute of Childrens Hospital Los Angeles, The Keck School of Medicine, University of Southern California, 4650 Sunset Blvd. MS #137, Los Angeles, CA, 90027, USA; 2 Department of Surgical and Radiological Sciences, University of California, Davis School of Veterinary Medicine, Davis, CA, 95616, USA; 3 Department of Cardiothoracic Surgery, Stanford University, Stanford, CA, 94305, USA

## Abstract

**Background:**

The use of porcine cells and organs as a source of xenografts for human patients would vastly increase the donor pool; however, both humans and Old World primates vigorously reject pig tissues due to xenoantibodies that react with the polysaccharide galactose α (1,3) galactose (αGal) present on the surface of many porcine cells. We previously examined the xenoantibody response in patients exposed to porcine hepatocytes via treatment(s) with bioartficial liver devices (BALs), composed of porcine cells in a support matrix. We determined that xenoantibodies in BAL-treated patients are predominantly directed at porcine αGal carbohydrate epitopes, and are encoded by a small number of germline heavy chain variable region (V_H_) immunoglobulin genes. The studies described in this manuscript were designed to identify whether the xenoantibody responses and the IgV_H _genes encoding antibodies to porcine hepatocytes in non-human primates used as preclinical models are similar to those in humans. Adult non-immunosuppressed rhesus monkeys (*Macaca mulatta*) were injected intra-portally with porcine hepatocytes or heterotopically transplanted with a porcine liver lobe. Peripheral blood leukocytes and serum were obtained prior to and at multiple time points after exposure, and the immune response was characterized, using ELISA to evaluate the levels and specificities of circulating xenoantibodies, and the production of cDNA libraries to determine the genes used by B cells to encode those antibodies.

**Results:**

Xenoantibodies produced following exposure to isolated hepatocytes and solid organ liver grafts were predominantly encoded by genes in the V_H_3 family, with a minor contribution from the V_H_4 family. Immunoglobulin heavy-chain gene (V_H_) cDNA library screening and gene sequencing of IgM libraries identified the genes as most closely-related to the IGHV3-11 and IGHV4-59 germline progenitors. One of the genes most similar to IGHV3-11, V_H_3-11^cyno^, has not been previously identified, and encodes xenoantibodies at later time points post-transplant. Sequencing of IgG clones revealed increased usage of the monkey germline progenitor most similar to human IGHV3-11 and the onset of mutations.

**Conclusion:**

The small number of IGV_H _genes encoding xenoantibodies to porcine hepatocytes in non-human primates and humans is highly conserved. Rhesus monkeys are an appropriate preclinical model for testing novel reagents such as those developed using structure-based drug design to target and deplete antibodies to porcine xenografts.

## Background

The use of porcine cells, tissues, and organs for transplantation or extracorporeal perfusion would greatly benefit the 86,000 patients on the United Network for Organ Sharing transplant waiting list, as well as those considered medically unsuitable for transplantation of scarce human organs or tissues [1]. Unfortunately, humans and Old World primates vigorously reject pig tissues due to xenoantibodies that react with the polysaccharide galactose α (1,3) galactose (αGal) present on the surface of many porcine cells. This rejection is the result of two processes, involving both preformed, circulating xenoantibodies, and those antibodies whose production is stimulated by the presence of the xenograft [2-4]. Despite this immunological barrier, porcine cells and tissues have been used clinically: pig heart valves have been utilized since 1967 [5], and islets have been transplanted into at least ten diabetic patients [6]. Numerous patients have also undergone extracorporeal perfusion using porcine livers to reduce circulating toxins [7,8], and new perfusion systems are continually being developed [9,10]. Bioartificial liver devices (BALs), containing porcine hepatocytes in a filter cartridge with a semi-permeable membrane, were first used in emergency situations, and have now entered clinical trials [11-14]. These BALs are primarily designed for treatment of acute liver failure as a "bridge" while awaiting a human liver graft for allotransplantation, or until the damaged liver recovers from injury [15].

We previously examined the xenoantibody response in patients exposed to porcine hepatocytes via treatment(s) with BALs [16]. We determined that xenoantibodies in BAL patients are predominantly directed at porcine αGal carbohydrate epitopes, and are encoded by a small number of germline heavy chain variable region (V_H_) immunoglobulin genes [17].

In an effort to define the nature of the immune response to individual hepatocytes and vascularized organ grafts in a pre-clinical lower primate model, we studied the xenoantibody response of rhesus monkeys (*Macaca mulatta*) to pig hepatocytes as isolated cells and as solid organ xenografts. This series of experiments allowed us to confirm that: [1] the immunoglobulin xenoantibody responses of non-immunosuppressed primates exposed to porcine hepatocytes were similar, if not identical, to the response elicited by a vascularized hepatic graft, and [2] both responses were encoded by alleles of the same germline progenitors as those utilized in humans exposed to pig hepatocytes via a BAL device. This new information suggests that the xenograft response in rhesus monkeys provides an appropriate model for the development of therapies for clinical application.

## Results

### Monkeys exposed to porcine hepatocytes show sustained and elevated xenoantibody levels directed at di-, tri- and penta-saccharide forms of the gal carbohydrate

**Figure 1:**
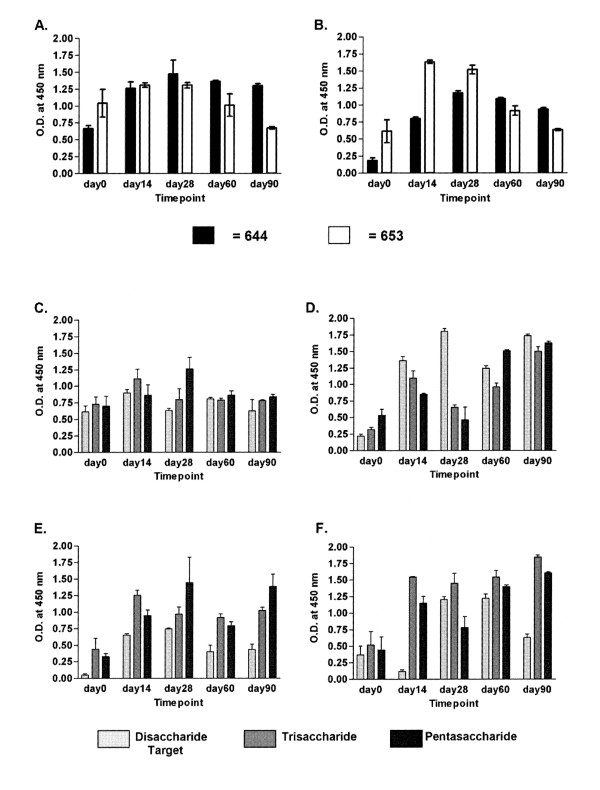
**ELISA assays demonstrating the induction of a xenoantibody response following infusion with porcine hepatocytes**. Two rhesus monkeys (#644 and #653) were infused with porcine hepatocytes, and the xenoantibody response was measured by ELISA. Panels (A.) and (B.) demonstrate the antibody response using pig aortic endothelial cells (PAEC) as antigenic targets. (A.) IgM and (B.) IgG. Panels (C.) through (F.) represent the anti-gal antibody response using purified di, tri, or penta αGal oligosaccharides as the antigenic target. (C.) #644 IgM. (D.) #653 IgM. (E.) #644 IgG. (F.) #653 IgG. Each sample was run in duplicate. Both IgM and IgG xenoantibodies against all tested antigenic targets were increased in each monkey post-porcine cell exposure.

Serum samples from monkeys infused with pig hepatocytes were tested to determine whether IgM and IgG xenoantibodies with specificity for αGal carbohydrate and for pig aortic endothelial (PAEC) xenoantigens were induced. As shown in Figure 1, there was an increase in binding of IgM (Fig. 1A.) and IgG (Fig. 1B.) xenoantibodies directed at pig endothelial antigens post-cell infusion. The increase in IgM levels from day 0 to day 14 in monkey #644 and the increase in pre and post infusion levels of IgG xenoantibodies were significant (p < 0.05). The day 14 timepoint was used to identify the immunoglobulin genes encoding xenoantibodies later in this study. These data indicated that the injection of pig hepatocytes was successful in eliciting humoral IgM and IgG xenoantibody responses. We also tested the serum from both animals to identify whether antibodies directed at the di-, tri- and penta-saccharide forms of αGal were induced (Figure 1 panels C through F). IgM (Figure 1C and 1E) and IgG (Figure 1D and 1F) levels against all three oligosaccharide forms of αGal increased after cell infusions. The increase in anti-gal IgM xenoantibodies shown from day 0 to day 14 were statistically significant for both monkeys. For the IgG response, (Figure 1F), monkey #653 demonstrated a significant increase in antibodies directed at gal tri- and pentasaccharides; the binding for the di-saccharide form was significant at PFI day 28.

**Figure 2:**
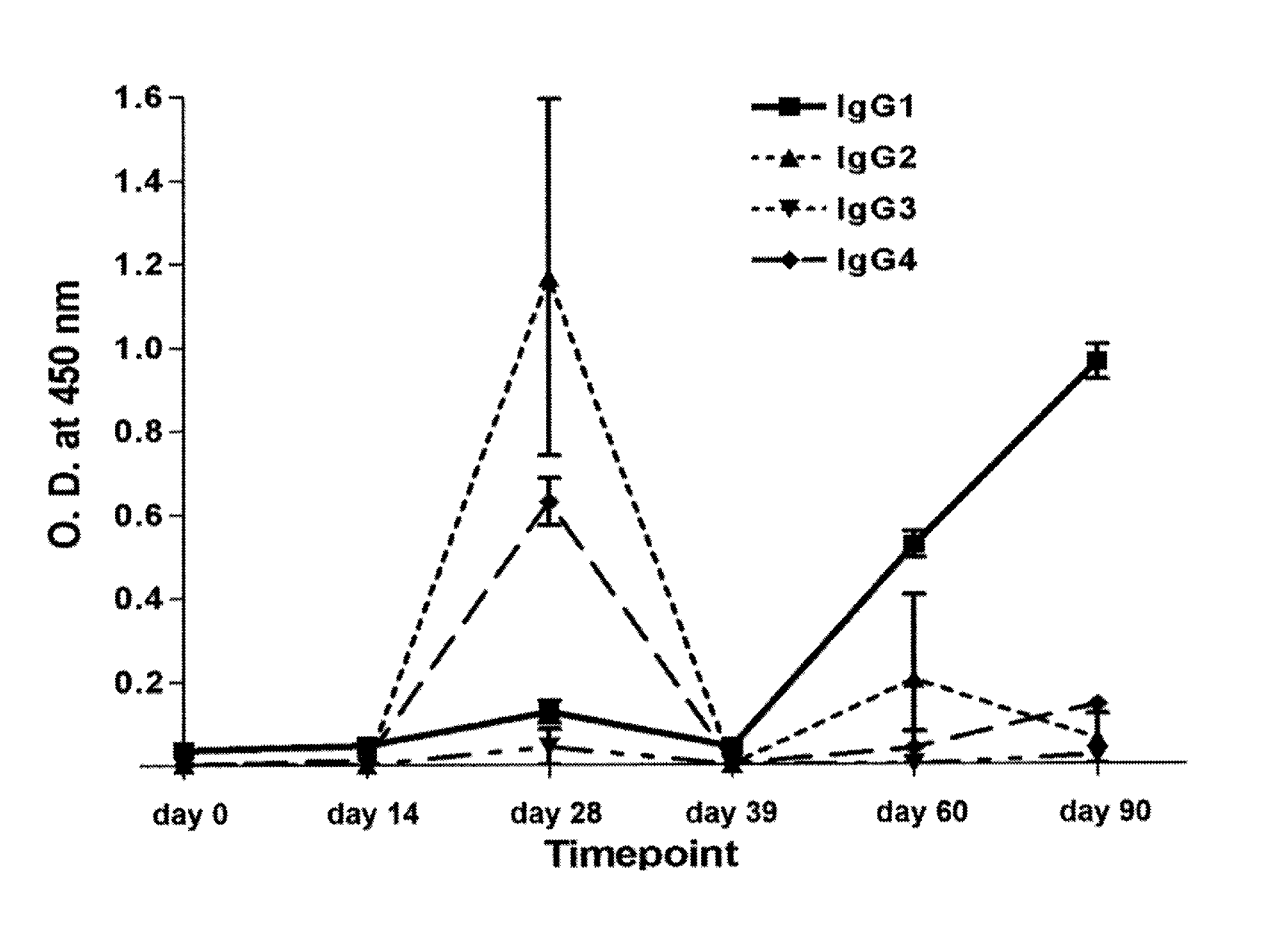
**IgG subclass expression with αGal pentasaccharide as the target antigen**. ELISA demonstrating an early increase in antibodies of the IgG2 and IgG4 **subclasses**. By PI (post-first injection) day 39, these antibodies decreased and a rise in IgG **subclass **1 levels was detected.

We then identified the subclass of IgG which were elevated during the xenoantibody response. As shown in Figure 2, there was an increase in IgG2 and IgG4 at day 28. IgG1 became significantly elevated later, and remained high for the duration of the study. The xenoantibody responses in rhesus monkeys are consistent with those seen in humans following exposure to porcine hepatocytes during the BAL procedure [16].

Despite the increase in anti-αGal IgM and IgG xenoantibody levels following pig hepatocyte exposure, we determined that there was very little fluctuation in the total levels of both IgM and IgG during the ninety-day course of the experiment as shown by ELISA using a standard curve (data not shown). Our results concur with other published data showing that the levels of total IgM and IgG can remain unchanged during the course of experiments in which anti-gal levels fluctuate due to xenoantibody depletion and xenoantibody return [18].

### Immunoglobulin gene expression was increased in the V_H_3 and V_H_4 families

**Figure 3:**
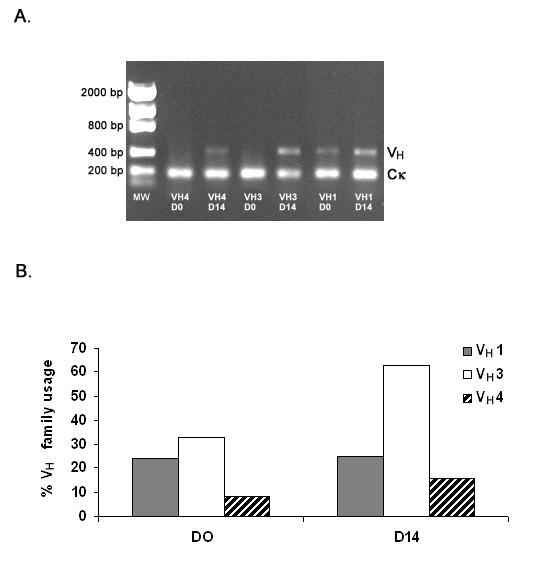
**The Ig repertoire post-exposure to porcine hepatocytes demonstrates increased usage of the V_H_3 and V_H_4 families**. Semiquantitative PCR (A) and colony filter hybridization (B) were used to demonstrate an increase in usage of the V_H_3 and V_H_4 family of genes at day 14 post-infusion with porcine hepatocytes.

Semi-quantitative PCR was initially performed to determine the Ig gene families in which elevated expression could be identified post-exposure to porcine hepatocytes. The results demonstrated that gene expression in the V_H_3 family was elevated, and a modest increase in V_H_4 expression was also identified (Figure 3A). These results were confirmed by colony filter hybridization using V_H _family specific primers to quantitate changes in immunoglobulin gene family usage prior to and following porcine hepatocyte exposure (Figure 3B). IgM libraries were then prepared to identify the germline progenitors encoding xenoantibodies in non-human primates mounting active xenoantibody responses to porcine hepatocytes. We screened for the presence of human genes that encode xenoantibodies [17,19], and IGHV3-11^cyno^, a cynomolgus monkey gene that closely matches an allele of IGHV3-11. This gene was expressed at high levels in cynomolgus monkeys that have been transplanted with transgenic porcine heart grafts (Zahorsky-Reeves, submitted).

We found that IGHV3-11 and IGHV3-11^cyno ^were up-regulated post-exposure in the V_H_3 IgM cDNA libraries prepared from both porcine-cell infused animals and liver lobe-transplanted animals, albeit with different kinetics (Table 1). The IGHV3-11 gene was expressed with greater frequency earlier in the course of the experiment, while IGHV3-11^cyno ^usage increased later. In hepatocyte-infused animals, IGHV3-11 expression rose by day 14 to as much as 20% of the V_H_3 PBL cDNA IgM library. By day 21, IGHV3-11^cyno ^expression was detected at high levels. Sequencing of at least 40 clones for each of the cDNA libraries indicated that no significant expression of any other V_H_3 germline progenitors was determined, including those V_H_3 genes shown to encode anti-αGal antibodies in normal individuals [19].

**Table 1:** Percent expression^1 ^of IGHV3-11^cyno ^and IGHV3-11 genes in IgM cDNA libraries

	Monkey #644		Monkey #653	
	IGHV3-11^cyno^	IGHV3-11	IGHV3-11^cyno^	IGHV3-11
Day 0 ^*a*^	9.4.	5.8	5.4	8.9
Day 14	12.0	19.0	6.3	20.0
Day 21	26.9	10.0	40.0	8.0
Day 28	6.1	5.7	18.0	4.0
Day 40 to 45	8.5	8.5	4.0	4.0

^1 ^= defined as the number of IGHV3-11^cyno ^and IGHV3-11 clones out of the total number of V_H_3 positive clones in each library. Between 100 to 300 V_H_3 positive colonies were represented in each library. ^*a *^= days are post-first injection (PI)

We then transplanted rhesus monkeys with a porcine liver lobe xenograft to determine whether IgV_H _genes encoding xenoantibodies induced following exposure to a solid organ graft are similar to those induced following exposure to isolated hepatocytes. A rapid increase in expression of both IGHV3-11 and IGHV3-11^cyno ^V_H_3 family genes was detected in the cDNA libraries post-porcine liver lobe transplantation. At four hours after establishment of circulation through the xenograft, the percent expression of both IGHV3-11 and IGHV3-11^cyno ^had dramatically increased, from 5% at the pre-transplant time point to 12.5%, as identified by colony filter hybridization. A marked increase in mRNA expression, occurring within 30 minutes post-stimulation of B cells, has similarly been reported in other studies [20,21]. The rapid increase in IGHV3-11 gene expression following exposure to the porcine liver may be due to the antigenic load of αGal epitopes, as it is postulated that there were far more endothelial cells and hepatocytes in the roughly 40 g solid liver lobe than the approximately 60 million individual cells given per hepatocyte infusion [2,22-24].

In order to identify the IgV_H _genes encoding xenoantibodies in the V_H_4 family, we prepared libraries using V_H_4 family-specific primers from the peripheral blood of monkeys at day 0 and at 14 days following porcine hepatocyte exposure. An increase in the expression of a monkey gene most similar to the human IGHV4-59 germline gene was demonstrated. The expression of this gene rose from 4.9% at day 0 to 9.8% at PI day 14, as determined by colony-filter hybridization, and confirmed by nucleic acid sequencing. Our laboratory recently reported that this germline progenitor showed an increase in expression in V_H_4 libraries of human BAL patients post-porcine cell exposure [25]. The results of our analysis indicate the IgV_H _gene usage in humans and rhesus monkeys is very similar following exposure to porcine hepatocytes.

### DNA sequences of IgVH genes encoding xenoantibodies in rhesus monkeys and humans are highly homologous

#### - DNA sequencing reveals high sequence identity to human IGHV3-11 and VH4-59 germline genes

**Figure 4:**
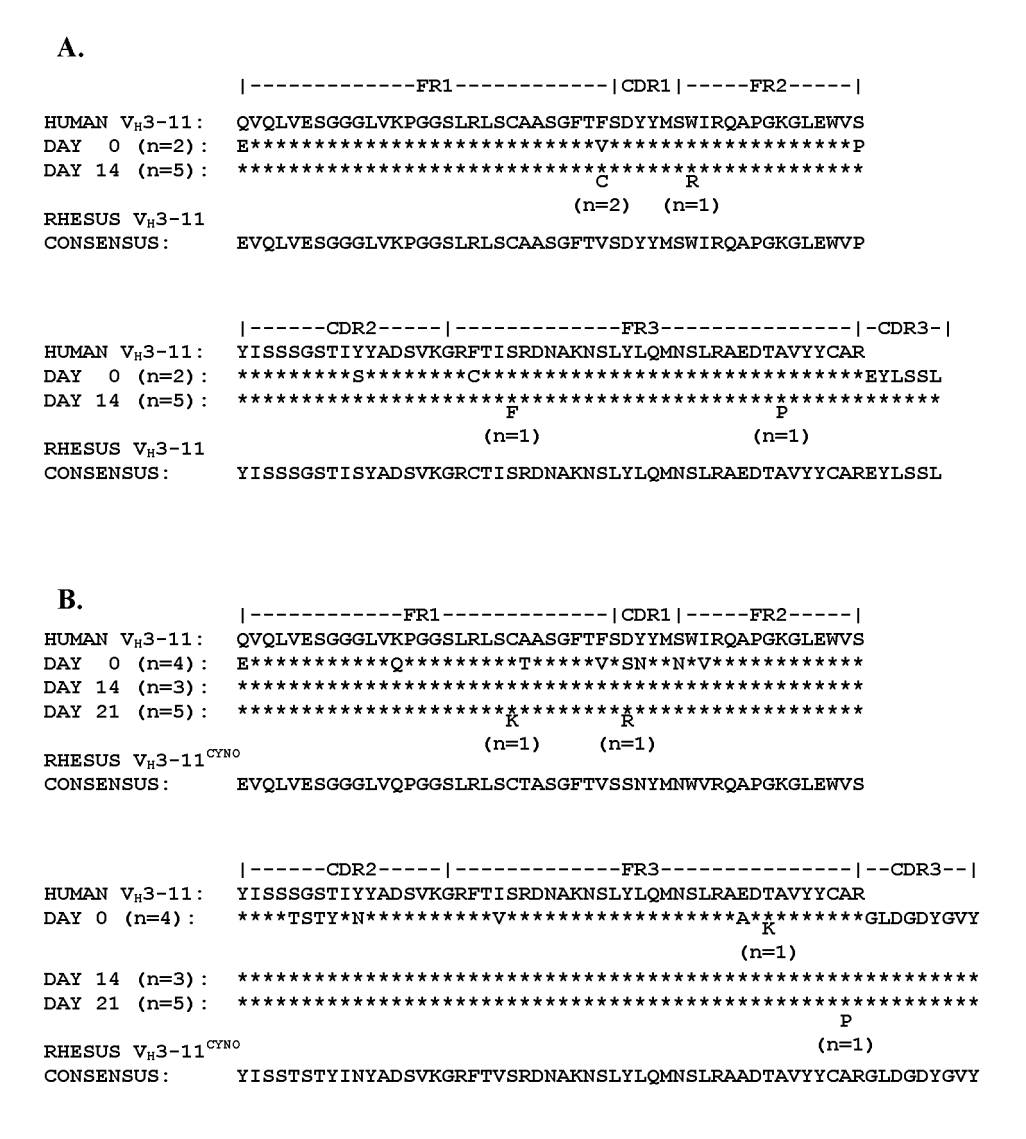
**Comparison of the derived amino acid sequences of IGHV3-11 (A.) and IGHV3-11^cyno ^(B.) **IgM genes encoding xenoantibodies in two porcine-cell infused animals, with reference to the human IGHV3-11 germline sequence from GenBank® (HSIGVH22B). * = residue is identical to one immediately above. Letters below the asterisks indicate amino acid substitutions.

We sequenced multiple IGHV3-11 and IGHV3-11^cyno^- positive clones from cDNA libraries prepared at several time points post-transplantation in all animals studied. The IgM amino acid sequences for the IGHV3-11 and IGHV3-11^cyno ^genes encoding xenoantibodies in the porcine-cell injected monkeys showed very few changes from day 0 to PI days 14 or 21 (Figure 4). The consensus nucleic acid sequence for the IGHV3-11 gene is 97% identical to human IGHV3-11 (the germline gene HSIGVH22B is the IGHV3-11 allele that encodes xenoantibodies in BAL treated humans [17]). The CDR3 "EYLSSL" amino acid sequence was associated with the IGHV3-11 allele HSIGVH22B in BAL treated humans [17]. As shown in Figure 4, the monkey IGHV3-11 sequences selected to encode xenoantibodies post-infusion used an identical CDR3. Monkey – specific segments of the Cμ and Cγ region of the cDNA clones, however, clearly distinguished these non-human primate sequences from their human counterparts.

The closest human immunoglobulin germline gene match for the consensus rhesus IGHV3-11^cyno ^gene is HSIGVH38, an allele of IGHV3-11, which shares 93% sequence identity. A ''TSTY'' amino acid motif in the CDR2 of IGHV3-11^cyno ^appears to be unique; the nucleic acids encoding this region do not match any known human or primate germline immunoglobulin genes in GenBank^® ^[26]. The IGHV3-11^cyno ^clones used the CDR3 segment ''GLDGDYGVY'', regardless of whether the xenograft exposure was due to individual pig cells or a liver lobe graft. This CDR3 was not found in association with any other V_H_3 gene that we have sequenced thus far from these IgM libraries.

**Figure 5:**
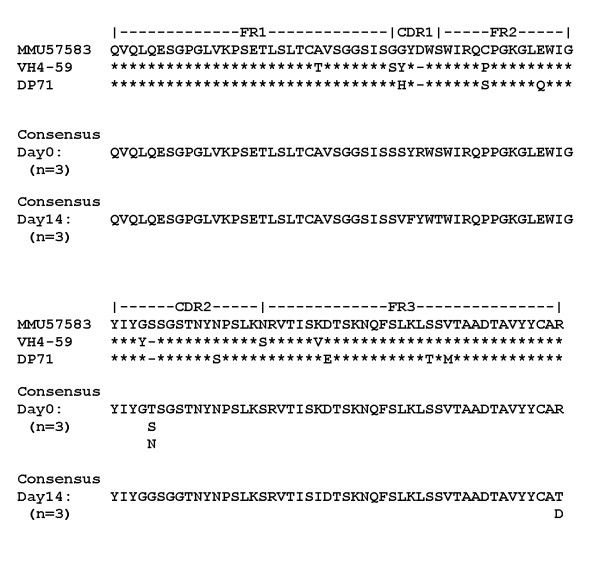
**Consensus amino acid comparisons of V_H_4 sequences encoding IgM xenoantibodies**. MMU57583 (top line) is a GenBank® macaque gene with high percent identity to many of the rhesus V_H_4 sequences. IGHV4-59 (second line) is the V_H_4 family gene shown to be increased post-pig cell exposure in a human BAL study. DP71 (third line) is an allele of IGHV4-59 to which many of the rhesus V_H_4 sequences match with high percent similarity. Consensus rhesus V_H_4 sequences at both days 0 and 14 show 89 to 92% nucleic acid sequence identity to DP71. * = residue is identical to one immediately above.

The sequence of the IgV_H _genes encoding xenoantibodies in the V_H_4 family were found to be most similar to the human IGHV4-59 germline progenitor. Sixteen clones were sequenced from the day 0 and day 14 IgM cDNA libraries. We generated a day 0 consensus nucleic acid sequence that showed a sequence similarity of 89% to an allele of human IGHV4-59, DP71 [26] and a 92% similarity to the macaque V_H_4 germline gene MMU5783 [27] (Figure 5). By PI day 14 there are two amino acid changes in the consensus sequence in the CDR1 region and one in the CDR2 region. The day 14 consensus sequence shares 92% nucleic sequence identity with the human DP71 germline gene. Genes encoding xenoantibodies in the V_H_4 family in rhesus monkeys are very similar to those identified in the human BAL study [17,25].

**Figure 6:**
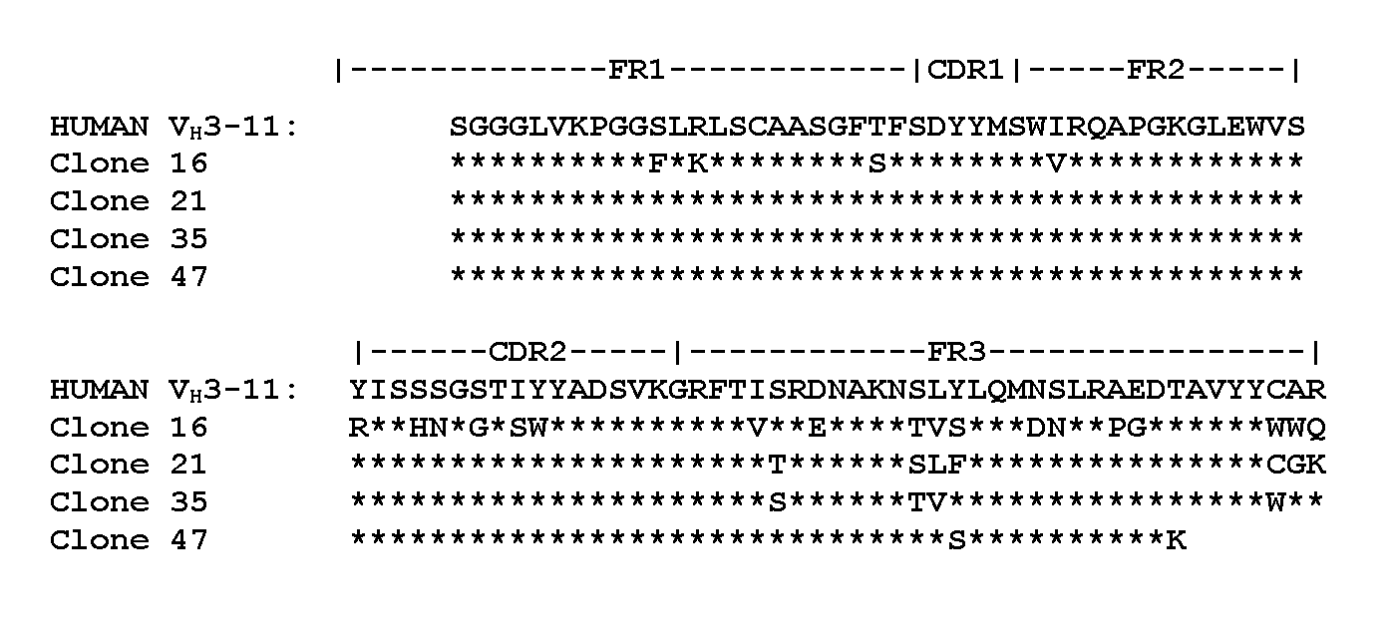
**Amino acid sequences of IGHV3-11 IgG clones**. Comparison of the derived amino acid sequences of IGHV3-11 IgG clones encoding xenoantibodies in two porcine-cell infused animals is shown with reference to the human IGHV3-11 germline sequence from GenBank®. * = residue is identical to one immediately above. Letters indicate amino acid substitutions.

By day 21, IgG xenoantibodies are expressed at high levels. Genes encoding IgG xenoantibodies were cloned and sequenced from the V_H_3 libraries prepared at day 0 and day 21 (Figure 6). Numerous mutations occurred in the IgG xenoantibodies expressed in these animals compared with the IgM clones that we had sequenced. The closest germline match, however, was to the human and rhesus IGHV3-11 germline progenitor. Similar mutations were also seen in human IGHV3-11 IgG sequences obtained from patients at 21 days after BAL treatment [17]. No clones corresponding to IGHV3-11^cyno ^were detected or sequenced from either timepoint in the IgG libraries.

## Discussion

Multiple experimental studies have led to the development of novel anti-rejection techniques designed to eliminate the hyperacute and acute xenograft rejection responses in lower primates by reducing or eliminating xenoantibody circulation and/or production [28-30]. Unfortunately, once treatment is terminated, xenoantibody levels rapidly return. If the genetic control of these antibodies were understood, the B cells encoding them could be targeted and potentially eliminated [31], ensuring the persistence of low circulating xenoantibody levels and improved graft survival in the transplant recipient. For example, a reduction of up to 87% of the cytotoxicity of baboon xenoantibodies was recently achieved, using anti-idiotypic antibodies generated against human anti-αGal antibodies [32]. Our laboratory has focused on understanding the structure of the xenoantibody binding pocket and the use of this information to apply computer-simulated drug design to identify novel drugs and reagents capable of preventing xenograft rejection.

In this study, we examined the humoral immunoglobulin response in rhesus monkeys injected with porcine hepatocytes, and compared this with the immune response following exposure to hepatocytes transplanted as a solid organ. We found no significant differences in the humoral response to hepatocytes expressed as solid organs or isolated cells. Although an analysis of the immune response in non-human primates placed on a BAL would have most closely mimicked our prior studies in human patients, this was not feasible within our experimental design.

We found a statistically significant elevation of xenoantibody levels directed at di-, tri- and pentasaccharide forms of the gal carbohydrate. These results are correlated with a previous study from our laboratory in which the immune responses of human patients treated with one or more BAL devices containing porcine hepatocytes was assessed [17]. Targets of this response included all forms of αGal. The subclasses of IgG xenoantibodies induced were similar to those previously reported in humans [16,33], indicating that an analogous immunoglobulin response is induced in humans and rhesus monkeys exposed to carbohydrate xenoantigens.

Xenoantibodies expressed by patients mounting an active humoral response are encoded by two V_H_3 family germline genes [17], which appear to be selectively expanded from a small number of genes that encode anti-αGal antibodies in naïve individuals [18]. In our present study, we detected a similar selective expansion of V_H_3 family genes that were homologous to those encoding xenoantibodies in the BAL-treated human patients [17]. Through nucleic acid sequencing of the IgM libraries, we were able to confirm that the genes encoding the majority of xenoantibodies were most closely-related to the human germline IGHV3-11 gene, while a small proportion of xenoantibodies are encoded by a gene most similar to the human IGHV4-59 germline gene. The IGHV3-11^cyno ^gene was not found to be elevated post-exposure in the human IgM or IgG libraries when examined up to 21 days post-BAL treatment. Single chain antibodies encoded by this gene, however, can bind to the gal carbohydrate as shown by the ability of these antibodies to partially block human natural antibody binding to gal (Zahorsky-Reeves et. al. submitted). The lack of expression of this gene in human patient samples may possibly be due to differences in the route of exposure to xenoantigens expressed on hepatocytes. BAL devices contain pores that limit exposure to porcine xenoantigens. Although the hepatocyte preparation we used for our study contained a combination of porcine hepatocytes and endothelial cells that was comparable to that found in a BAL device (MultiCell Technologies, private communication), the route of exposure may account for the fact that the IGHV3-11^cyno ^gene was not induced in the study in human patients.

In humans, IgM V_H _genes encoding xenoantibodies induced following xenoantibody exposure were expressed in germline configuration [17]. A separate study involving the spectrotypic analysis of human anti-αGal antibodies reported similar results [34]. This is in agreement with prior work in our lab demonstrating that genes expressed in germline configuration encode xenoantibodies in several small animal models of xenograft rejection [35-37]. Comparison of nucleic acid sequences to known germline genes in rhesus monkeys indicates that the closest non-human primate germline gene is a monkey homolog of IGHV3-11 (clone 18, AF173920). Since the discovery and reporting of rhesus monkey germline immunoglobulin genes is still in the early stages, there are currently insufficient numbers of nucleic acid sequences for germline progenitors at this time to conclusively determine whether or not the IgM xenoantibodies in rhesus monkeys are expressed in germline configuration [26]. The conservation in the sequence and structure of the genes encoding xenoantibodies in humans and non-human primates, however, suggests that the unique binding pocket formed by these IgM natural antibodies have an optimal affinity for carbohydrate xenoantigens.

The structural features of the xenoantibody/gal binding pocket and the role of the CDR3 in binding specificity for gal and other carbohydrates are currently under investigation in our laboratory. The CDR3 region is important as both its sequence and its length can be indicative of the level of diversity in the genetic repertoire [38,39], and it may play a direct role in the formation of the antigen-specific binding site [39]. Approximately 95% of all IGHV3-11 clones sequenced from these monkeys used the same CDR3 as that used by the human patients in the BAL study [17]. Additionally, 100% of the IGHV3-11^cyno ^clones sequenced post-exposure used the same CDR3 sequence, which was different than that associated with the IGHV3-11 gene. The study that had examined the genes used by naïve humans encoding anti-αGal antibodies found the CDR3 region to be highly divergent in both composition and length [18]. Our results suggest that a particular combination of V_H _gene and CDR3 is effective in binding with sufficient affinity to αGal epitopes to be selected for further expansion. The relative affinities of the IGHV3-11^cyno ^and IGHV3-11 gene products to αGal and/or other structurally-related carbohydrates should provide further insight into the molecular basis for the selective usage of these genes encoding xenoantibodies.

## Conclusion

Our findings show that rhesus monkeys transplanted with hepatocytes expressed as cells or as a solid organ xenograft develop a humoral response encoded by similar genes as those encoding humoral xenograft responses in human patients [17]. This consistency of response will allow for the development of clinical therapies in lower primates that can directly be transferred to humans.

## Methods

### Animals

All work was approved by both the Animal Care and Use Committee of the California Regional Primate Research Center (CRPRC) at the University of California, Davis, and the Institutional Animal Care and Use Committee (IACUC) of the University of Southern California (USC). Young adult male captive-bred rhesus monkeys (*Macaca mulatta*) were obtained from the CRPRC primate colony, where the animals were housed and all surgical and sampling procedures were conducted. We prescreened monkeys by ELISA and selected those with relatively low baseline levels of xenoantibody for these studies.

### Porcine cell infusion

Primary pig hepatocytes, containing approximately 7% endothelial cells [40], were a kind gift from MultiCell Technologies, Inc. (Providence, Rhode Island, USA). Sixty million cells were washed, pelleted, and aseptically suspended in 20 cc of sterile normal saline. A surgical midline abdominal approach was performed on two monkeys, aged 20 months (#644) and 42 months (#653). In each monkey, hepatocytes were slowly infused into the portal circulation via a jejunal vein catheter [41]. Each monkey recovered without complications. This procedure was repeated twice in each animal, at days 14 and 28 post infusion (PI). Blood samples were taken at multiple time points until PI day 90.

### Porcine partial liver lobe implantation

The liver and its accompanying vessels were isolated from a 10 day-old Yorkshire-cross male piglet. A midline abdominal incision was made into a 4.4 year old rhesus monkey (#785). The liver was flushed *in situ *with chilled preservation solution (Viaspan™, DuPont Pharma, Wilmington, Delaware, USA). The approximately 40 g graft was harvested from the left liver lobe with intact portal triad vessels. This graft was implanted heterotopically into the infrahepatic region of the primate by anastomosing the graft vena cava end-to-side to the primate's inferior vena cava, and the grafts' superior mesenteric vein to the right side of the primate's portal vein. The graft's aortal segment was implanted laterally on the infra-renal aorta of the recipient and unclamped, achieving full revascularization. The graft became discolored and congested within two hours of graft placement, indicating hyperacute rejection.

### ELISA : Quantitation of whole IgM and IgG

We wished to verify that, over time, there was a steady level of antibodies present in the monkeys' serum, such that dilution of 1:20 for all time points would contain approximately the same concentration (at ng/ml) of immunoglobulin. We used human IgM and IgG ELISA Quantitation Kits (Bethyl Laboratories Inc, Montgomery, Texas, USA), as the antibodies in these kits cross-react with rhesus serum, according to the manufacturer. Serum samples were run in duplicate at 1:20 and compared with a standard curve of known human serum antibodies.

### ELISA : Anti-pig aortic endothelial cell (PAEC) binding

ELISAs were used for assessment of both IgM and IgG binding as previously developed [42]. Serum samples from porcine-cell infused monkeys from day 0 and at PI days 10, 14, 28, 60 and 90 were used, with a naïive human serum sample as a control. Briefly, 96-well microtiter plates were coated with fixed PAEC and frozen at -80°C until use. Thawed plates were washed and blocked routinely with 1% bovine serum albumin (BSA). The plates were washed after incubation of the serum at room temperature for 1 hour. Secondary antibody was applied at appropriate dilutions: peroxidase-conjugated AffiniPure F(ab')2 fragment goat anti-human IgM from Jackson ImmunoResearch (catalog #109-036-129) (West Grove, Pennsylvania, USA) or peroxidase-labeled goat anti-human IgG (γ-chain specific), F(ab')_2_fragment, from Sigma (catalog # A2290) (St. Louis, Missouri, USA). After one hour, the plates were again washed and the substrate (Sure Blue™, KPL, Gaithersburg, Maryland, USA) applied. After color development, the reaction was stopped by addition of H_2_SO_4_. Each plate was promptly read on a Perkin Elmer HTS 7000 Plus BioAssay Reader at 450 nm. Data were organized and analyzed using a standard t test on Prism software.

### ELISA : anti-αGal xenoantibody binding

Serum from porcine-cell infused animals (PI days 0, 14, 28, 60, and 90) were tested for anti-αGal xenoantibodies by ELISA using plates coated with the di-, tri- and penta-saccharide forms of αGal at 0.25 μg/well (Dextra Laboratories, Reading, UK). The protocol was as described for the anti-PAEC assay.

### ELISA : IgG subclasses

We determined the distribution of the IgG subclasses expressed in three rhesus monkeys over the course of their immune responses. Serum from PI days 0, 14, 28, 39, 60 and 90 were used from porcine-cell infused monkeys, and run in duplicate. Final assays were done using serum in the absence of additional dilution ("neat") and naïve human serum as a positive control. Briefly, 96-well microtiter plates were coated overnight with Galα1-3Galα1-4GlcNAc (αGal pentasaccharide) (Dextra). Blocking and serum incubation steps were done as described above. Sheep anti-human IgG secondary antibodies were added (The Binding Site, San Diego, California, USA) at the following dilutions: anti-IgG_1 _(catalog #AP006) at 1:400 dilution; anti-IgG_2 _(catalog #AP007) at 1:100; anti-IgG_3 _(catalog #AP008) at 1:300 and anti-IgG_4 _(catalog #AP009) at 1:100, and then incubated for one hour at room temperature. These anti-subclass IgG antibodies have all, with the exception of the anti-human IgG3, been shown to cross-react with macaque IgG immunoglobulins [43-45]. After final washes, peroxidase substrate color reaction, plate reading and data analysis were performed as described above.

### cDNA preparation

Peripheral blood leukocytes (PBLs) were extracted at the CRPRC from whole blood samples, flash frozen and stored at -80°C. From these samples of approximately 1 to 5 million cells, RNA was routinely extracted (QIAGEN RNeasy Kit, QIAGEN, Valencia, California, USA). Naïve human PBL samples were prepared in tandem as positive controls. Double-stranded cDNA was synthesized from the RNA, using techniques that had been successful previously for human samples (cDNA Synthesis Kit, Roche, Basel, Switzerland) [17] and then purified using Microcon 100 columns (Amicon, Millipore, Billerica, Massachusetts, USA).

### Library construction

cDNA libraries of genes encoding IgM antibodies [17] were constructed from PBLs isolated from porcine-cell infused monkeys at PI days 0, 10, 14, 21, and 28; libraries were also created at day 0, post surgery, and at sacrifice (24 hours) for the liver lobe transplanted animal (#785). PCRs for amplification of both families were performed as previously described [17]. PCR products were purified and ligated into a pCR^® ^2.1 vector (Original TA Cloning Kit, Invitrogen, Carlsbad, California, USA). Ligation reactions were transformed into INVαF' One Shot™ Competent Cells (Invitrogen) and plated onto Xgal-containing LB ampicillin plates. Genes encoding IgG xenoantibodies from the IgV_H _family 3 were cloned from days 0 and 21 using PCR and the primers Monk CGI 5'-GGGTTGTAGTCC-TTGACCAGGCAG-3' and primer Monk CGII 5'GACCGATGGGCCC TTGGTGGAGGC-3', both specific for the constant region of IgG. The last PCR reaction was visualized on a 2% ethidium bromide agarose gel. The band closest to the predicted size of 425 bp was cut, eluted by electrolysis with 0.5× TBE-buffer, cloned (Original TA Cloning Kit, Invitrogen, Carlsbad, CA), and transformed as above.

### Semi-quantitative PCR

An analysis of the V_H _family repertoire in rhesus monkeys prior to and following porcine xenoantigen exposure was done by semi-quantitative PCR using the following V_H _family specific 5' primers that recognize untranslated leader sequences: V_H_1, ATGGACTGGACCTGG; V_H_2, ATACTTTGTTCCACGCTCCT; V_H_3, GAGTTTGGGCTGAGCTGG; V_H_4, CTGGTGGCAGCTCCCAGA, V_H_5 ATCCTCGCCCTCCTCCTAGC, and V_H_6 TGTCTCCTTCCTCATCTTCC. These V_H _leader sequences identify immunoglobulin genes in non-human primates (27,46). The 3' primer (AGGAGAATTCTGAGGAGACGGTGACCAGGGT) was based on a consensus sequence for germline J_H _genes and has been previously used for semi-quantitative analysis of immunoglobulin family repertoire usage in rhesus monkeys (46). The Cκ gene provided an internal control and was amplified using the primers:5' ACCAAGGTCGACATCAAACGAACTGTGGCT and 3' CTGTCTAGCTCTGTGACACTCTCCTGGAG. These primers cross-react with macaque and human immunoglobulin genes (47,48). PCR runs were performed for 30 cycles (94°C for 20sec, 58°C for 30sec, and 72°C for 30 sec) in a Perkin-Elmer Gene Amp 9600 Thermal Cycler. Five pmol of V_H _and J_H _primers were used in the reaction along with 2.5 pmol of Cκ primers. Reaction products were visualized on an agarose gel and quantitated using BIORAD Quantity One Software, Version 4.0.3 (BIORAD, Hercules, CA). The V_H _family signals were normalized using the Cκ gene (46).

### Screening

V_H _family-specific leader primers (described above) were labeled with digoxigenin and used in colony filter hybridization experiments to confirm changes in the V_H _family repertoire in the pre and post-exposure peripheral blood samples. For this purpose, immunoglobulin gene libraries were amplified using Cμ and anchor primers, as previously published (17). Colony lifts were performed using nylon membranes (Roche) and a chemiluminescent (digoxigenin) detection protocol (DIG easy Hyb, Roche) [17]. Filters were hybridized with labeled probes and positive colonies were counted to determine the relative percentage of colonies specific for each V_H _gene family in the cDNA libraries. DNA sequencing was done on selected colonies to confirm the specificity of the probes. In addition to the V_H _family-specific leader probes, oligonucleotide probes used for this study also included: the RVH11 (5'TCACTTTCAGTGACTACTACATGAGCTGGA3') probe that is specific for the CDR1 region of human germline VH3-11, the probe 193 (5' AGTACTACAAACTATGCGG') for the CDR2 region of germline VH3-74 and its alleles (HSIGDP53 and HSIGCOS6), the probe 583IC (5'TAGTTATGAAATGAACT3') for the CDR1 region of germline HSIGDP58, the probe 543IC (5'AACATAAAGCAAGATGGA3') for the CDR2 region of germline VH3-7; and the probe (5'ATTGGGTATATCTATTACAGTGGGAGCACCAAC3') for the CDR2 region of germline VH4-59 [17]. These probes also included an oligonucleotide (called CYNO2, with the sequence 5'CATTAGTAGTACTAGTACTTACATAAACTACGC3') that was previously designed in our laboratory to detect a CDR2 region specific for a particular monkey V_H_3 gene (designated V_H_3-11^cyno^). This gene was expressed with high frequency post-exposure in cynomolgus monkeys transplanted with transgenic porcine heart grafts (Zahorsky-Reeves, submitted).

### DNA sequencing

Clones selected on the basis of colony filter hybridization, or a minimum of 40 clones from each library (IgM and IgG), were randomly chosen, grown overnight, prepared using the QIAPrep Spin MiniPrep Kit (QIAGEN) and sequenced using the ALFexpress™ automated DNA sequencer and the AutoCycle™ Sequencing Kit (Pharmacia Biotech, Piscataway, New Jersey, USA). Sequences obtained included the region from framework 1 (FR1) through complementarity determining region 3 (CDR3). Results were analyzed using OMIGA software and the closest identifiable germline counterparts in the GenBank^® ^library was determined using BLAST. Nucleotide sequence data reported are available in the GenBank database under the accession numbers [DQ023238 through DQ023262 and 986391 through 986394].

## Abbreviations

αGal = galactose α (1,3) galactose

BAL = bioartificial liver device

CDR = complementarity determining region

FR = framework

hDAF = human decay accelerating factor

PI = post-first infusion

V_H _= variable-region heavy chain

## Authors' contributions

JZR performed the ELISA assays, prepared, screened and sequenced the IgM cDNA libraries, and wrote the manuscript; CRG performed the monkey surgeries, in collaboration with AEK and DCB; DVC initiated the design for the project, interpreted data, and edited the manuscript; IYP performed the IgG library preparations and screening, including DNA sequencing of clones from this library; MKKJ provided scientific and experimental guidance throughout the study and edited the manuscript. All authors read and approved the final manuscript.
